# The effect of Selenium on thyroid physiology and pathology

**DOI:** 10.1186/1756-6614-8-S1-A23

**Published:** 2015-06-22

**Authors:** Elżbieta Skowrońska-Jóźwiak

**Affiliations:** 1 Department of Endocrinology and Metabolic Diseases, Medical University of Lodz, Polish Mother’s Memorial Hospital – Research Institute, Lodz, Poland

## 

Selenium (Se) is an important trace element for human physiology. It has anti-inflammatory, anti-neoplastic and anti-aging properties and protects from oxidative stress [1]. It is present in muscles, liver and kidneys, but reaches its highest concentration in the thyroid gland [2]. Selenium is incorporated in the molecular structure of a class of proteins called selenoproteins and the following well-characterized selenoproteins are found in the thyroid gland: glutathione peroxidases and thioredoxin reductases, protecting thyroid against free radicals, and deiodinases type I and II, participating in the synthesis of thyroid hormones [2]. Selenium status appears to have an impact on the development of several thyroid pathologies; autoimmune thyroid diseases, including Hashimoto disease and Graves-Basedow disease, thyroid orbitopathy, goiter, nodules and thyroid cancer.

Low concentrations of Se were shown in patients with Graves' disease [3]. Higher serum Se levels were seen in patients who went into remission and remained euthyroid during a 2-year follow-up period when compared with patients who did not receive permanent euthyreosis [4]. Results of these studies encourage the implementation of supplemental Se in the treatment of hyperthyroidism together with antithyroid drugs as well as to continue further research into new selenium-rich thyrostatics. The clinical study GRASS comparing the effectiveness of thyrotoxicosis treatment with thyrostatics alone and that accompanied by Se is just being carried out [5]. There are some data about the role of Se in the treatment of thyroid orbitopathy. Serum Se levels are lower in patients with orbitopathy when compared with Graves' disease patients without orbitopathy [6]. Selenium supplementation has proven to prevent deterioration of mild Graves' ophthalmopathy [7]. In the single study Se was shown to be effective in the prevention of the post-partum surge of TPO-Ab and thyroid dysfunction [8], however the data about Se effect in Hashimoto disease are conflicting; according to the Summary of a Cochrane Systematic Review the evidence to support or refute the efficacy of Se supplementation in people with Hashimoto's thyroiditis is incomplete and not reliable to help in clinical decision making [9].

The influence of Se on goitrogenesis and carcinogenesis in the thyroid gland is still unclear. The data on effect of low Se levels in patients with goiter and thyroid nodules are divergent [1,10]. In vitro study showed that Se is able to decrease thyroid cancer growth [11]. In one study Se was inversely correlated with stages of thyroid cancer [12], but in a post-diagnosis study there was no association between fingernail selenium levels and thyroid cancer risk [13].

Conclusive results about the undisputable role of Se in the treatment of thyroid related diseases, including hyperthyroidism or thyroid orbitopathy, on a wider scale are still unavailable and further research is both required and recommended.
